# Effect of non-pharmacological interventions on pain in preterm infants in the neonatal intensive care unit: a network meta-analysis of randomized controlled trials

**DOI:** 10.1186/s12887-023-04488-y

**Published:** 2024-01-03

**Authors:** Yuwei Weng, Jie Zhang, Zhifang Chen

**Affiliations:** 1 Medical School of Nantong University, Nantong, 226001 China; 2 Obstetrical Department, Affiliated Maternity and Child Health Care Hospital of Nantong University, Nantong, 226001 China

**Keywords:** Non-pharmacological intervention, Neonatal intensive care unit, Preterm infant, Pain, Network meta-analysis

## Abstract

**Objective:**

To evaluate the effectiveness of different non-pharmacological interventions for pain management in preterm infants and provide high-quality clinical evidence.

**Methods:**

Randomized controlled trials (RCTs) of various non-pharmacological interventions for pain management in preterm infants were searched from PubMed, Web of Science, Embase, and the Cochrane Library from 2000 to the present (updated March 2023). The primary outcome was pain score reported as standardized mean difference (SMD). The secondary outcomes were oxygen saturation and heart rate reported as the same form.

**Results:**

Thirty five RCTs of 2134 preterm infants were included in the meta-analysis, involving 6 interventions: olfactory stimulation, combined oral sucrose and non-nutritive sucking (OS + NNS), facilitated tucking, auditory intervention, tactile relief, and mixed intervention. Based on moderate-quality evidence, OS + NNS (OR: 3.92, 95% CI: 1.72, 6.15, SUCRA score: 0.73), facilitated tucking (OR: 2.51, 95% CI: 1.15, 3.90, SUCRA score: 0.29), auditory intervention (OR: 2.48, 95% CI: 0.91, 4.10, SUCRA score: 0.27), olfactory stimulation (OR: 1.80, 95% CI: 0.51, 3.14, SUCRA score: 0.25), and mixed intervention (OR: 2.26, 95% CI: 0.10, 4.38, SUCRA score: 0.14) were all superior to the control group for pain relief. For oxygen saturation, facilitated tucking (OR: 1.94, 95% CI: 0.66, 3.35, SUCRA score: 0.64) and auditory intervention (OR: 1.04, 95% CI: 0.22, 2.04, SUCRA score: 0.36) were superior to the control. For heart rate, none of the comparisons between the various interventions were statistically significant.

**Conclusion:**

This study showed that there are notable variations in the effectiveness of different non-pharmacological interventions in terms of pain scores and oxygen saturation. However, there was no evidence of any improvement in heart rate.

**Supplementary Information:**

The online version contains supplementary material available at 10.1186/s12887-023-04488-y.

## Introduction

Preterm infants in the neonatal intensive care unit (NICU) frequently undergo painful procedures such as venipuncture, heel-stick, and endotracheal suctioning, as well as orogastric tube insertion [1]. The pain and stress from frequent procedures can have both transient and enduring impacts on the behavior, physiology, and neurodevelopment of preterm infants [2]. Research indicated that at 7 years of age, preterm children who underwent more invasive neonatal procedures had higher salivary cortisol levels and internalizing behavior scores greater than full-term children [3]. Another research found that cumulative pain and stress were associated with neurobehavioral outcomes such as stress/abstinence and habituation responses in preterm infants [4]. Reports from South Korea, Canada and Kenya indicated that many preterm infants continued to receive highly invasive procedures without adequate analgesia, highlighting an ongoing need to improve pain management practices in this vulnerable population [5–8].

Given the suboptimal pain management practices and risk of adverse outcomes demonstrated in preterm infants, there is growing interest in identifying and evaluating effective analgesic interventions for this population. However, a review reported that commonly used anesthetic and sedative agents may have both acute and long-term detrimental neurological impacts in preterm infants [9]. The American Academy of Pediatrics also stated that the long-term effects and safety of pharmacologic analgesia are yet to be studied [10]. Clearly, there is a need to explore alternative, neuroprotective pain management strategies in this vulnerable population. In recent years, non-pharmacological interventions such as skin-to-skin contact, non-nutritive sucking, facilitated tucking position, breastfeeding, oral sucrose, olfactory stimulation, and music therapy have emerged as effective methods for pain management in preterm infants [11, 12]. Evidence has already confirmed their efficacy and safety in pain management and some other pain-related indicators such as oxygen saturation, respiratory rate, and heart rate [13–18].

Previous systematic reviews have primarily examined the effectiveness of individual or combined non-pharmacologic interventions for the treatment of pain in preterm infants [13–15, 17, 18]. While there have been several recent systematic reviews assessing the effectiveness of different non-pharmacological interventions, it is important to note that these reviews have not encompassed the entire spectrum of interventions, and the evidence has not been consolidated [11, 16, 19, 20]. As such, the objective of this network meta-analysis is to integrate various non-pharmacological interventions and evaluate their efficacy in managing pain in preterm infants, providing high-quality clinical evidence for improving pain care.

## Methods

### Search strategy

The review was conducted and reported following the PRISMA guidelines [21]. The protocol for this meta-analysis has been registered in the PROSPERO database (CRD42023412200). We searched PubMed, Web of Science, Embase, and the Cochrane Library for randomized controlled trials (RCTs) from 2000 to the present (updated March 2023) using the targeted search strategy provided in the Data Supplement (Additional file 1: Appendix Table 1). The search was restricted to English articles. The search strategy used both medical subject heading terms and keywords for pain, infant, preterm, neonatal intensive care unit and so on.

### Study selection

**Fig. 1:**
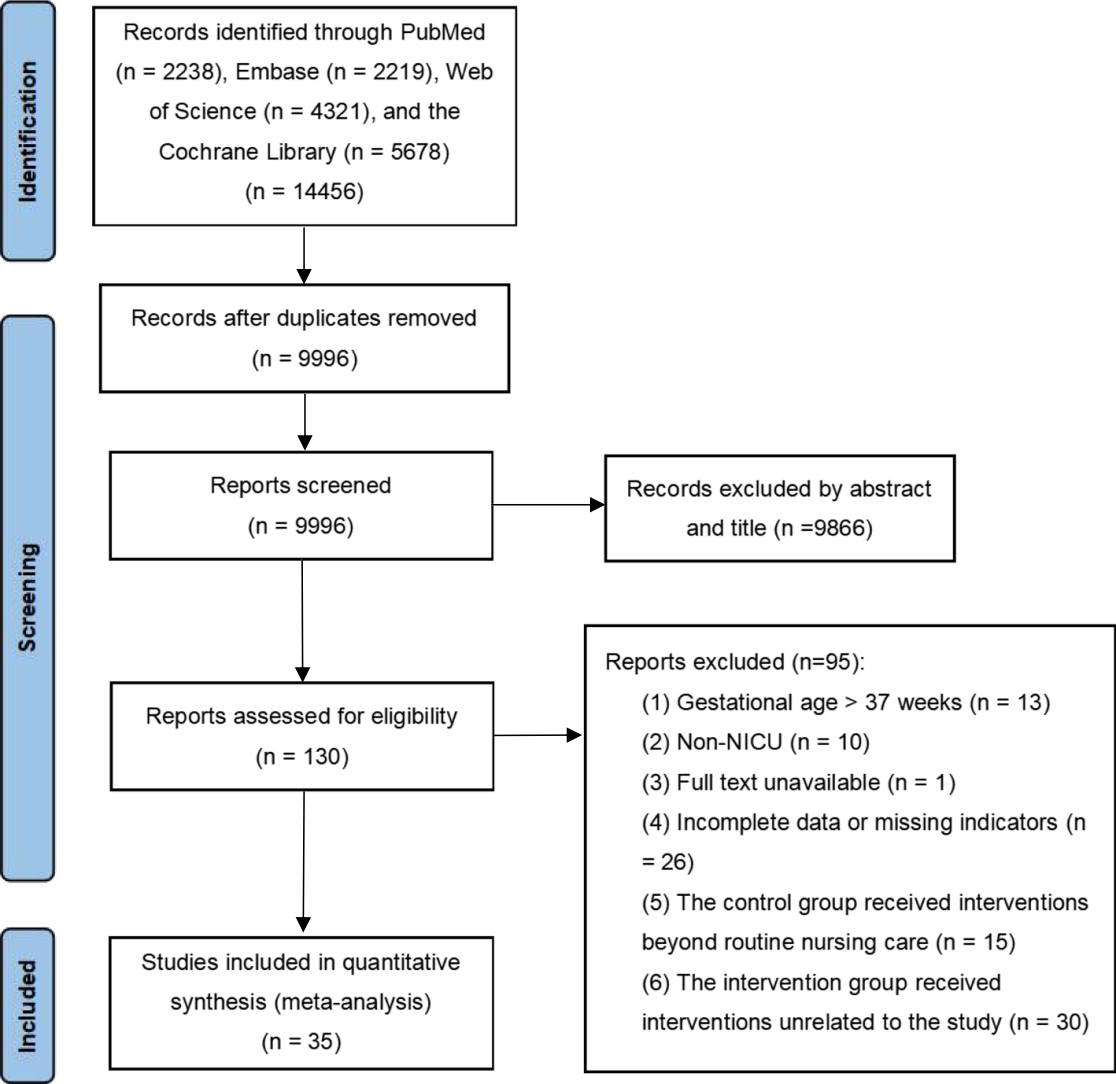
Flow diagram of study inclusion and exclusion

Two authors (Yuwei Weng and Jie Zhang) independently evaluated the articles to determine their eligibility for inclusion, and differences were addressed by agreement. The eligible full texts were reviewed after they were screened for titles and abstracts (Fig. 1). The criteria for inclusion were as follows: (1) The participants were preterm infants in the NICU (gestational age < 37 weeks). (2) Studies were RCTs. (3) The experimental group implemented tactile relief (Kangaroo mother care, massage, etc.), auditory intervention (mothers’ voice, white noise, lullaby, etc.), olfactory stimulation (maternal breast milk odor, vanilla odor, amniotic fluid odor, etc.), combined oral sucrose and non-nutritive sucking (OS + NNS), facilitated tucking, or mixed intervention. (4) The control group received routine nursing care, including placebo, pacifier, and incubator. The criteria for exclusion were as follows: (1) Full-term infants or other non-preterm infants. (2) Results of pain score, oxygen saturation and heart rate were ambiguous or missing. (3) Non-RCTs, non-English literature, non-human studies, repeated publications, reviews, and meta-analyses were excluded.

### Data extraction

Two authors (Yuwei Weng and Jie Zhang) independently reviewed the article and extracted relevant data and parameters. The EndNote X9 software was used to import all the retrieved articles, and duplicates were removed. After a preliminary selection of titles and abstracts, the remaining eligible articles were checked for full text according to the prespecified inclusion and exclusion criteria. When the eligible articles were reviewed, the following parameters were extracted: first author, publication year, RCT design, participants, intervention and control groups (sample size), gestational age and birth weight, painful procedures, and outcomes. Any discrepancies were resolved with the assistance of the third author (Zhifang Chen) throughout the entire process of study search, article review, and data extraction.

### Quality assessment

To assess the risk of bias in the included RCTs, two authors (Yuwei Weng and Jie Zhang) independently used the Cochrane Risk of Bias Assessment Tool [22]. This tool evaluated six aspects of the studies, namely selection bias, performance bias, detection bias, attrition bias, reporting bias, and other bias. Each aspect was evaluated through one or more items and was classified as low, high, or unclear risk. Due to the large number of interventions and articles involved in this meta-analysis, if there was any disagreement in the evaluation process, consensus would be reached through discussion. In addition, the certainty of evidence was assessed using the Confidence in Network Meta-Analysis (CINEMA) [23] framework, which comprises six domains: within-study bias, reporting bias, indirectness, imprecision, heterogeneity, and incoherence. The certainty of the results was graded as high, moderate, low, or very low.

### Outcomes

The main outcome was pain score. The extraction of pain data was based on the last time node. Data were expressed as continuous variables (SMD). The results of the pain score were evaluated using the Premature Infant Pain Profifile (PIPP) [24]. The scale is a tool designed to assess pain in preterm infants who are between 28 to 36 weeks of gestation. It consists of seven items, which are further categorized into three behavioral items, two physiological items, and two contextual items. The scale was revised and promoted in 2014 to improve its accuracy and sensitivity in consideration of psychometric properties for extremely low gestational age (ELGA) infants and feedback from clinical medical staff on the percentage calculation problem [25]. The scale measures pain on a range of 0 to 21, with higher scores indicating more significant pain. The secondary outcomes measured were oxygen saturation and heart rate. The form of data presentation and the time nodes extracted were consistent with the pain score.

### Statistical analysis

Standardized mean differences were initially chosen based on the expression of continuous variables. Following this, the Cochran’s Q statistic and the I^2^ statistic were used to explore the heterogeneity among studies. The random-effects model was chosen when there was heterogeneity (I^2^ ≥ 50%). Otherwise, the fixed-effects model was chosen [26, 27]. Afterwords, the efficacy of various interventions was assessed using network meta-analysis, with the consistency of direct and indirect comparisons being evaluated using the loop inconsistency test, and the efficacy ranking of the interventions was observed. Subsequent to the main analysis, sensitivity analyses were carried out and the possibility of publication bias was evaluated through the use of funnel plots. Ultimately, all statistical assessments were conducted with the aid of Stata version 17.0 and the gemte package in R software [28].

## Results

### Study selection

A comprehensive search was conducted on PubMed, Web of Science, Embase, and the Cochrane Library, resulting in the identification of 14,456 publications. After removing duplicates, 9,996 publications were reviewed. Through preliminary screening of titles and abstracts, 130 studies were identified that focused on non-pharmacological interventions for pain in preterm infants. Of these, 1 was excluded because the full text was unavailable. After reviewing the remaining 129 publications, 13 were excluded due to the fact that the study subjects were not preterm infants. Additionally, 10 publications were excluded because the study sites were not NICUs. 26 publications were excluded due to incomplete or missing outcome data, while 15 were excluded because the control group received non-routine care. Therefore, 35 RCTs of 2134 preterm infants included at the end [29–63]. The PRISMA flow diagram of the included studies is presented in Fig. 1.

Table 1 presents the categorization of the studies based on their characteristics. The studies included in this analysis were published between 2000 to the present (updated March 2023), and the sample size ranged from 20 [35] to 200 [57]. The mean gestational age of preterm infants varied between 26 [45, 60] to 37 [31, 38, 58, 63] weeks, while the mean birth weight ranged from 932.3 [62] to 2,299.03 [30] grams. In addition, the design of RCTs, painful procedures, details of grouping, and outcomes for all studies were also summarized in Table 1.

**Table 1:** Characteristics of included studies

First author, year	RCT design	Participants/Intervention/Control (sample size)	Gestational age(week)/Birth weight(g)	Painful procedures	Outcomes
Baudesson de Chaville, 2017 [34]	Double-blind, placebo-controlled, RCT, two parallel groups	G1: MBMO (*n* = 16) G2: control: an odorless diffuser (*n* = 17)	Total: 33.2 (31.6–34.1) Total: 1790 (1647–1947)	Venipuncture	PIPP score
Jebreili 2015 [44]	RCT, three parallel groups	G1: MBMO (*n* = 45) G2: vanilla odor (*n* = 45) G3: control: routine nursing care (*n* = 45)	G1: 31.64 ± 2.1/1,566.9 ± 414.89 G2: 30.93 ± 2 /1,505.3 ± 409.12 G3: 31.46 ± 1.96 /1,569.8 ± 405.93	Venipuncture	PIPP score
Alemdar, 2017 [48]	RCT, four parallel groups	G1: amniotic fluid odor (*n* = 21) G2: MBMO (*n* = 22) G3: mother odor (*n* = 20) G4: control: routine nursing care (*n* = 22)	G1: 33.95 ± 3.20 /2,235.04 ± 801.76 G2: 32.09 ± 3.42 /1,939.00 ± 836.78 G3: 33.05 ± 3.17 /2,120.10 ± 797.15 G4: 33.40 ± 3.11 /2,193.06 ± 679.80	Heel-stick	PIPP score Heart rate Oxygen saturation
Alemdar, 2020 [30]	RCT, two parallel groups	G1: amniotic fluid odor (*n* = 30) G2: control: routine nursing care (*n* = 31)	G1: 31.30 ± 2.57 /1,734.73 ± 599.04 G2: 33.90 ± 3.17 /2,299.03 ± 758.21	Peripheral cannulation	PIPP score
Usta, 2021 [61]	Double-blind, placebo-controlled, RCT, two parallel groups	G1: lavender oil odor (*n* = 31) G2: control: distilled, odorless water (*n* = 30)	G1: 32.45 ± 2.29 /1834.45 ± 448.51 G2: 33.10 ± 2.75 /1961.93 ± 522.82	Heel lance	PIPP-R score
Rad, 2021 [55]	Single-blind, placebo-controlled, RCT, three parallel groups	G1: MBMO (*n* = 30) G2: another mother’s breast milk odor (*n* = 30) G3: control: distilled water (*n* = 30)	G1: 32.9 ± 2.4 /1806 ± 553 G2: 30.3 ± 3.2 /1620 ± 425 G3: 32.5 ± 2.4 /1688 ± 404	HBV injection	PIPP score Heart rate Oxygen saturation
Asmerom, 2013 [33]	Double-blind, RCT, three parallel groups	G1: OS + NNS (*n* = 44) G2: sterile water + NNS (*n* = 45) G3: control: routine nursing care (*n* = 42)	G1: 30.1 ± 3.1 /1374.1 ± 552 G2: 31.5 ± 2.1 /1498.4 ± 706 G3: 30.5 ± 2.6 /1456.4 ± 502	Heel lance	PIPP score Heart rate Oxygen saturation
Dilli, 2014 [37]	Placebo-controlled, RCT, two parallel groups	G1: OS + NNS (*n* = 32) G2: control: sterile water + pacifier (*n* = 32)	G1: 28.2 ± 2.7 /1248 ± 392 G2: 28.8 ± 2.9 /1360 ± 530	ROP screening	PIPP score
Gao, 2018 [41]	RCT, four parallel groups	G1: OS (*n* = 21) G2: NNS (*n* = 22) G3: OS + NNS (*n* = 22) G4: control: routine nursing care (*n* = 21)	G1: 31.7 ± 0.9 /1780.8 ± 304.6 G2: 31.9 ± 1.1 /1767.3 ± 302.7 G3: 32.0 ± 0.8 /1697.1 ± 254.7 G4: 31.3 ± 0.6 /1682.7 ± 200.2	Heel-stick	PIPP score Heart rate Oxygen saturation
Liaw, 2012 [51]	RCT, three cross-over groups	G1: FT (*n* = 34) G2: NNS (*n* = 34) G3: control: routine nursing care (*n* = 34)	Total: 33.98 ± 2.0 Total: 1705.9 ± 363.3	Heel-stick	PIPP score
Sundaram, 2013 [59]	Single-blind, RCT, two cross-over groups	G1: FT (*n* = 20) G2: control: routine nursing care (*n* = 20)	Total: 34.11 ± 2.29 Total: 2153 ± 532.84	Heel-stick	PIPP score
Alinejad-Naeini, 2014 [31]	RCT, two cross-over groups	G1: FT (*n* = 34) G2: control: routine nursing care (*n* = 34)	Total: 29 ~ 37 weeks Total: ≥ 1200 g	Endotracheal suctioning	PIPP score
Hill, 2005 [42]	RCT, two cross-over groups	G1: FT (*n* = 12) G2: control: routine nursing care (*n* = 12)	Total: 28.8 ± 2.8 Total: 1410 ± 473	Routine care (nasogastric tube insertion)	PIPP score
Ward-Larson, 2004 [62]	RCT, two cross-over groups	G1: FT (*n* = 40) G2: control: routine nursing care (*n* = 40)	Total: 27.313 ± 2.430 Total: 932.30 ± 284.05	Endotracheal suctioning	PIPP score
Davari, 2019 [36]	RCT, two cross-over groups	G1: FT (*n* = 40) G2: control: routine nursing care (*n* = 40)	Total: 32 ~ 36 weeks Total: ≥ 1200 g	Heel-stick	PIPP score
Apaydin, 2020 [32]	RCT, six parallel groups	G1: swaddling (*n* = 30) G2: FT (*n* = 32) G3: EBM (*n* = 31) G4: swaddling + EBM (*n* = 30) G5: FT + EBM (*n* = 31) G6: control: routine nursing care (*n* = 33)	Total: 33.11 ± 0.84 Total: 1989.41 ± 369.51	Orogastric tube insertion	PIPP score Heart rate Oxygen saturation
Döra, 2021 [38]	RCT, three parallel groups	G1: white noise (*n* = 22) G2: lullaby (*n* = 22) G3: control: routine nursing care (*n* = 22)	Total: 32 ~ 37 weeks Total: ≥ 1001 g	Venous blood collection	PIPP score Heart rate Oxygen saturation
Yu, 2022 [63]	Double-blind, RCT, two parallel groups	G1: maternal heart sounds (*n* = 32) G2: control: routine nursing care (*n* = 32)	Total: < 37 weeks Total: 1860.92 ± 506.26	Heel-stick	Heart rate Oxygen saturation
Kahraman, 2020 [47]	RCT, four parallel groups	G1: white noise (*n* = 16) G2: mothers’ voice (*n* = 16) G3: MiniMuffs (*n* = 16) G4: control: routine nursing care (*n* = 16)	G1: 33.8 ± 1.75 /1909 ± 340 G2: 34.0 ± 1.50 /1904 ± 325 G3: 34.06 ± 1.76 /2186 ± 621 G4: 34.25 ± 1.65 /2201 ± 615	Heel lance	Heart rate Oxygen saturation
Kurdahi Badr, 2017 [50]	Double-blind, RCT, three cross-over groups	G1: lullaby (*n* = 42) G2: mother's music (*n* = 42) G3: control: routine nursing care + headphones (*n* = 42)	Total: 31.78 ± 2.8 Total: 1577 ± 499.2	Heel-stick	Heart rate Oxygen saturation
Kucukoglu, 2016 [49]	RCT, two parallel groups	G1: white noise (*n* = 35) G2: control: routine nursing care (*n* = 40)	G1: 31.77 ± 3.30 /1673.29 ± 321.16 G2: 31.30 ± 2.50 /1530.62 ± 347.25	HBV injection	PIPP score Heart rate Oxygen saturation
Taplak, 2021 [60]	RCT, four parallel groups	G1: BMO (*n* = 20) G2: white noise (*n* = 20) G3: FT (*n* = 20) G4: control: routine nursing care (*n* = 20)	Total: 26 ~ 35.6 weeks Total: ≤ 1500 g (*n* = 38), ≥ 1501 g (*n* = 42)	Endotracheal suctioning	PIPP-R score Heart rate Oxygen saturation
Alemdar, 2018 [29]	RCT, four parallel groups	G1: BMO (*n* = 32) G2: maternal voice (*n* = 30) G3: incubator cover (*n* = 31) G4: control: routine nursing care (*n* = 32)	G1: 30.26 ± 0.69 /1430.70 ± 146.00 G2: 30.06 ± 0.63 /1460.50 ± 133.36 G3: 30.22 ± 0.66 /1404.80 ± 99.23 G4: 30.25 ± 0.50 /1503.80 ± 194.86	Peripheral cannulation	PIPP score
Mitchell, 2013 [52]	RCT, two parallel groups	G1: KC (*n* = 19) G2: control: routine nursing care (*n* = 19)	Total: 27 ~ 30 weeks G1: 1311.5 ± 216.3 G2: 1213.2 ± 186.4	Routine care (suctioning via tracheal or nasal routes)	PIPP score
Cong, 2011 [35]	RCT, two cross-over groups	G1: KC (*n* = 10) G2: control: routine nursing care (*n* = 10)	Total: 30 ~ 32 weeks Total: 1577 ± 327	Heel-stick	PIPP score
Srivastava, 2022 [58]	Open label, RCT, two parallel groups	G1: KMC (*n* = 40) G2: control: routine nursing care (*n* = 40)	Total: 28 ~ 37 weeks Total: 1500-2499 g	Orogastric tube insertion	PIPP-R score
Johnston, 2008 [46]	Single-blind, RCT, two cross-over groups	G1: KMC (*n* = 61) G2: control: routine nursing care (*n* = 61)	Total: 28 0/7 ~ 31 0/7 weeks	Heel lance	PIPP score
Johnston, 2013 [45]	RCT, two parallel groups	G1: therapeutic touch (*n* = 27) G2: control: routine nursing care (*n* = 28)	Total: 26 0/7 ~ 28 6/7 weeks G1: 974.54 ± 188 G2: 977.44 ± 210	Heel lance	PIPP score
Fatollahzade, 2022 [40]	RCT, two cross-over groups	G1: GHT (*n* = 34) G2: control: routine nursing care (*n* = 34)	Total: 27 ~ 34 weeks Total: ≥ 1200 g	Endotracheal suctioning	PIPP score
Sezer Efe, 2022 [56]	Assessor-blind, RCT, two parallel groups	G1: GHT (*n* = 25) G2: control: routine nursing care (*n* = 25)	G1: 34.95 ± 1.61 /2272.70 ± 430.19 G2: 35.3 ± 1.83 /2289.37 ± 630.80	Heel lance	Heart rate Oxygen saturation
Dur, 2020 [39]	RCT, three parallel groups	G1: GHT (*n* = 30) G2: Yakson touch (*n* = 30) G3: control: routine nursing care + pacifier (*n* = 30)	Total: 33.44 ± 1.74 Total: 1960.83 ± 413.75	Heel lance	Heart rate Oxygen saturation
Jain, 2006 [43]	Double-blind, RCT, two cross-over groups	G1: massage (*n* = 23) G2: control: routine nursing care (*n* = 23)	Total: 31.1 ± 1.9 Total: 1693 ± 396	Heel-stick	Heart rate Oxygen saturation
Qiu, 2017 [54]	RCT, two parallel groups	G1: music + GHT (*n* = 30) G2: control: routine nursing care (*n* = 32)	G1: 34.30 ± 0.67 /1930 ± 130 G2: 33.33 ± 0.54 /2000 ± 70	Routine care (tracheal aspiration, nasal aspiration, removal of intravenous lines, etc.)	PIPP score
Shukla, 2018 [57]	Double-blind, RCT, four parallel groups	G1: KMC (*n* = 50) G2: music therapy (*n* = 49) G3: KMC + music therapy (*n* = 50) G4: control: routine nursing care (*n* = 51)	Total: 34.0 ± 2.32 Total: 1910 ± 340	Heel prick	PIPP score
Perroteau 2018 [53]	RCT, two parallel groups	G1: FT + NNS (*n* = 30) G2: control: routine nursing care + pacifier (*n* = 29)	Total: 29.0 (28.0–31.0) Total: 1300.0 (1130.0–1530.0)	Heel-stick	PIPP score

*MBMO* Maternal breast milk odor, *BMO* Breast milk odor, *EBM* Expressed breast milk, *OS* Oral sucrose, *NNS* Non-nutritive sucking, *FT* Facilitated tucking, *KC* Kangaroo care, *KMC* Kangaroo mother care, *GHT* Gentle human touch

### Risk of bias in included studies

**Fig. 2:**
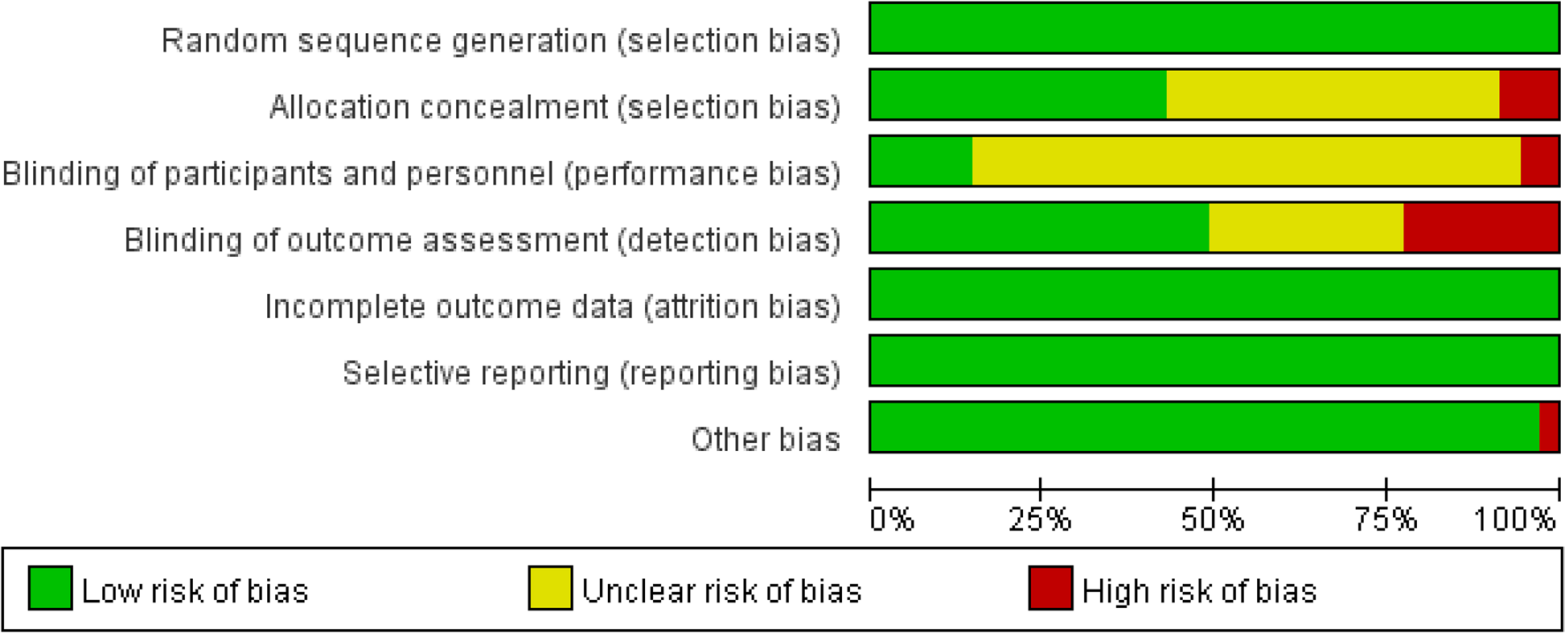
Risk of bias graph

Figure 2 reported the results of the bias risk assessment for the included studies. Of the allocation concealment methods, 3 studies [53, 56, 58] were marked as high risk for not applying, 17 [29–31, 36, 39–42, 44, 46, 48, 49, 51, 52, 54, 55, 62] were marked as unclear risk for not being mentioned in the study, and the remaining were marked as low risk. In the blinding of participants and personnel, 2 studies [58, 59] took a high risk since they couldn’t apply the blinding, and 28 [29–32, 35, 36, 38–42, 44–57, 60, 62, 63] were marked as unclear risk. In the blinding of outcome assessment, 8 studies [29, 30, 33, 35, 47, 48, 51, 52] were found to be marked as high risk for not applying the blinding and 10 [31, 36–39, 41, 42, 58, 61, 62] were marked as unclear risk. Among other bias, 1 study [56] was marked as high risk as the study personnel and outcome assessment was the same person. In addition, all studies explained the use of randomization methods and were accordingly marked as low risk. Incomplete outcome data and selective reporting were also not found in the studies.

### PIPP scores

**Fig. 3:**
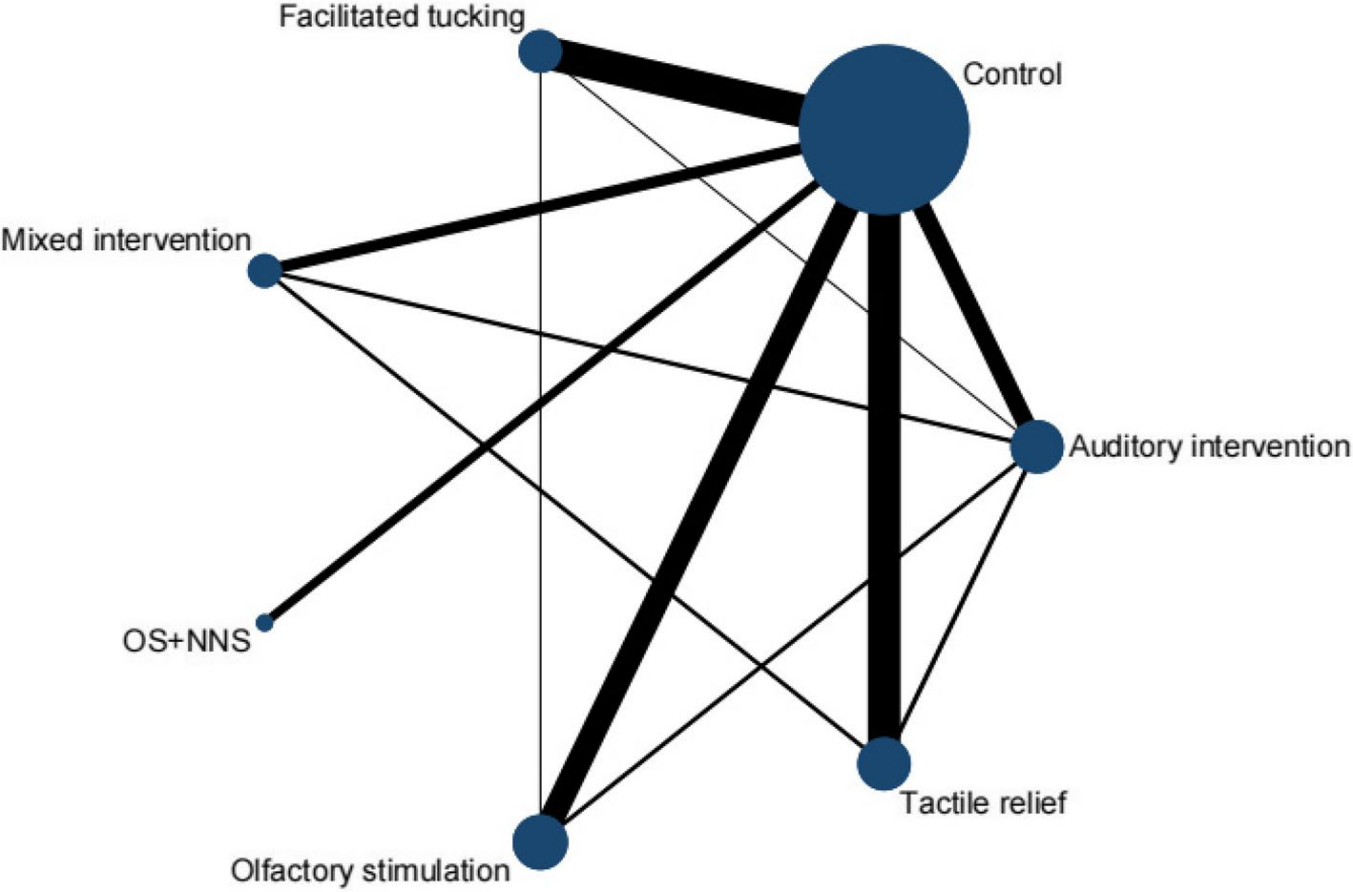
Network evidence diagram (PIPP score)

A total of 29 RCTs [29–38, 40–42, 44–46, 48, 49, 51–55, 57–62] were included in this meta-analysis by PIPP score, involving 6 interventions. Olfactory stimulation (8 RCTs), OS + NNS (3 RCTs), facilitated tucking (8 RCTs), auditory intervention (5 RCTs), tactile relief (7 RCTs), and mixed intervention (3 RCTs) were included. A total of 7 nodes were included in this meta-analysis, with each node representing an intervention or control (Fig. 3). The nodes with more significant interactions were control (34 interactions), olfactory stimulation (11 interactions), auditory intervention (10 interactions), facilitated tucking (9 interactions), and tactile relief (8 interactions). The results of the consistency analysis was shown in Additional file 1: Appendix Table 2. The results of the heterogeneity test indicated a high degree of heterogeneity with an I^2^ value of 97.1%.

**Fig. 4:**
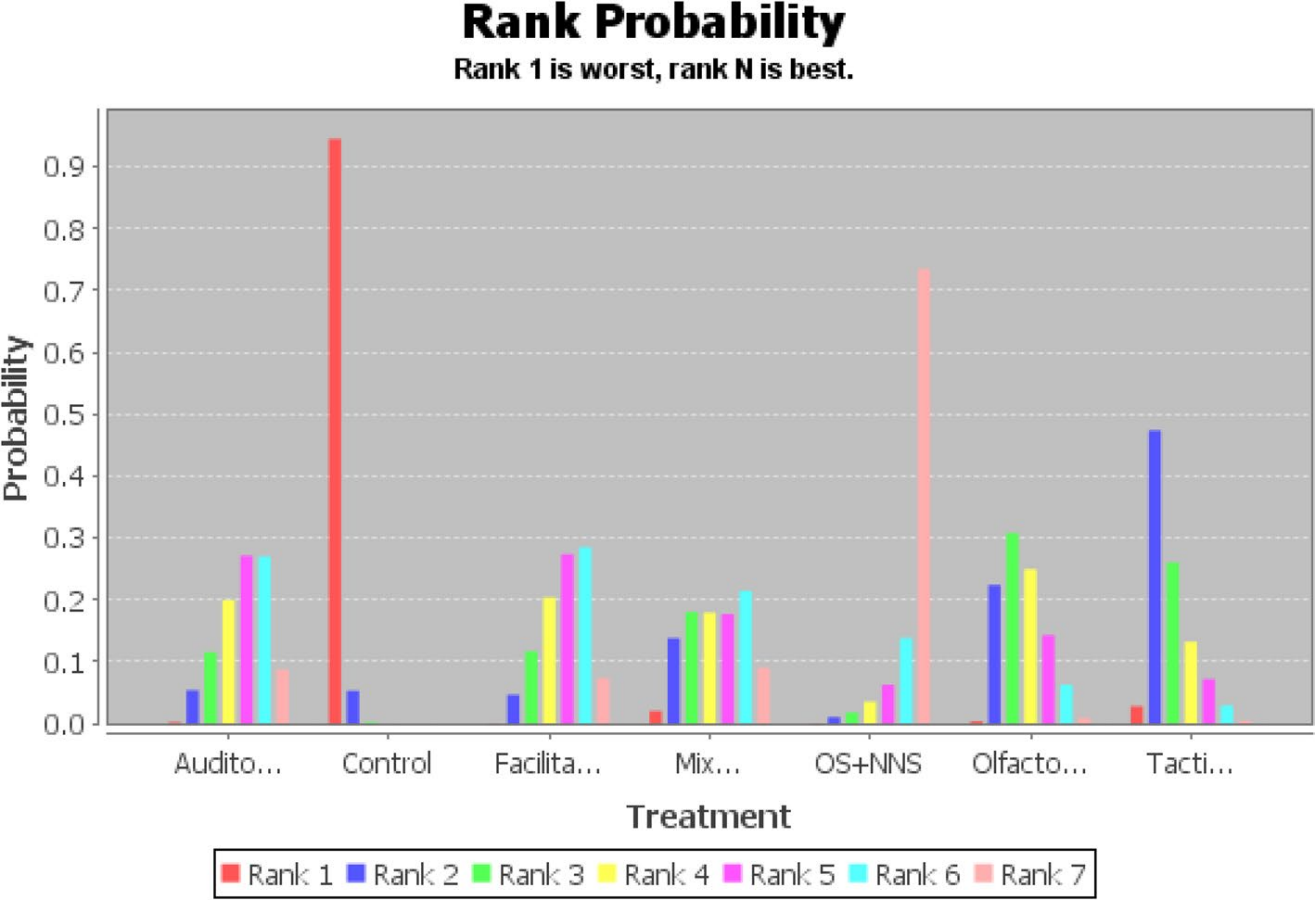
Probability ranking diagram of best interventions (PIPP score)

Based on moderate-quality evidence (Additional file 1: Appendix Table 5), OS + NNS had the greatest SUCRA score, followed by facilitated tucking, auditory intervention, olfactory stimulation, tactile relief, mixed intervention, and control group (Fig. 4 and Additional file 1: Appendix Table 3). Compared to the control group, OS + NNS was 3.92 (95% CI: 1.72,6.15, SUCRA score: 0.73) lower, facilitated tucking was 2.51 (95% CI: 1.15,3.90, SUCRA score: 0.29) lower, auditory intervention was 2.48 (95% CI: 0.91. 4.10, SUCRA score: 0.27) lower, olfactory stimulation was 1.80 (95% CI:0.51,3.14, SUCRA score: 0.25) lower, and mixed intervention was 2.26 (95% CI:0.10,4.38, SUCRA score: 0.14) lower (Table 2). This study found that the comparison between tactile relief and the control group wasn’t statistically significant (OR: 1.38, 95% CI: -0.11,2.87, SUCRA score: 0.26). Therefore, the complete probability ranking was OS + NNS (73%) > facilitated tucking (29%) > auditory intervention (27%) > olfactory stimulation (25%) > mixed intervention (14%) > control group (94%) (Additional file 1: Appendix Table 3). The stability and credibility of the results were demonstrated in Additional file 1: Appendix Table 4 through the sensitivity analysis.

**Table 2:** The results of network meta-analysis (consistency model, PIPP score)

Auditory intervention	2.48 (0.91, 4.10)	-0.04 (-2.03, 2.01)	0.22 (-2.33, 2.77)	-1.45 (-4.16, 1.31)	0.68 (-1.19, 2.56)	1.10 (-1.00, 3.22)
-2.48 (-4.10, -0.91)	Control	-2.51 (-3.90, -1.15)	-2.26 (-4.38, -0.10)	-3.92 (-6.15, -1.72)	-1.80 (-3.14, -0.51)	-1.38 (-2.87, 0.11)
0.04 (-2.01, 2.03)	2.51 (1.15, 3.90)	Facilitated tucking	0.25 (-2.25, 2.78)	-1.41 (-4.02, 1.23)	0.70 (-1.15, 2.50)	1.14 (-0.95, 3.10)
-0.22 (-2.77, 2.33)	2.26 (0.10, 4.38)	-0.25 (-2.78, 2.25)	Mixed intervention	-1.66 (-4.78, 1.40)	0.45 (-2.04, 2.95)	0.88 (-1.61, 3.32)
1.45 (-1.31, 4.16)	3.92 (1.72, 6.15)	1.41 (-1.23, 4.02)	1.66 (-1.40, 4.78)	OS + NNS	2.11 (-0.52, 4.72)	2.53 (-0.06, 5.25)
-0.68 (-2.56, 1.19)	1.80 (0.51, 3.14)	-0.70 (-2.50, 1.15)	-0.45 (-2.95, 2.04)	-2.11 (-4.72, 0.52)	Olfactory stimulation	0.43 (-1.55, 2.43)
-1.10 (-3.22, 1.00)	1.38 (-0.11, 2.87)	-1.14 (-3.10, 0.95)	-0.88 (-3.32, 1.61)	-2.53 (-5.25, 0.06)	-0.43 (-2.43, 1.55)	Tactile relief

### Oxygen saturation

**Fig. 5:**
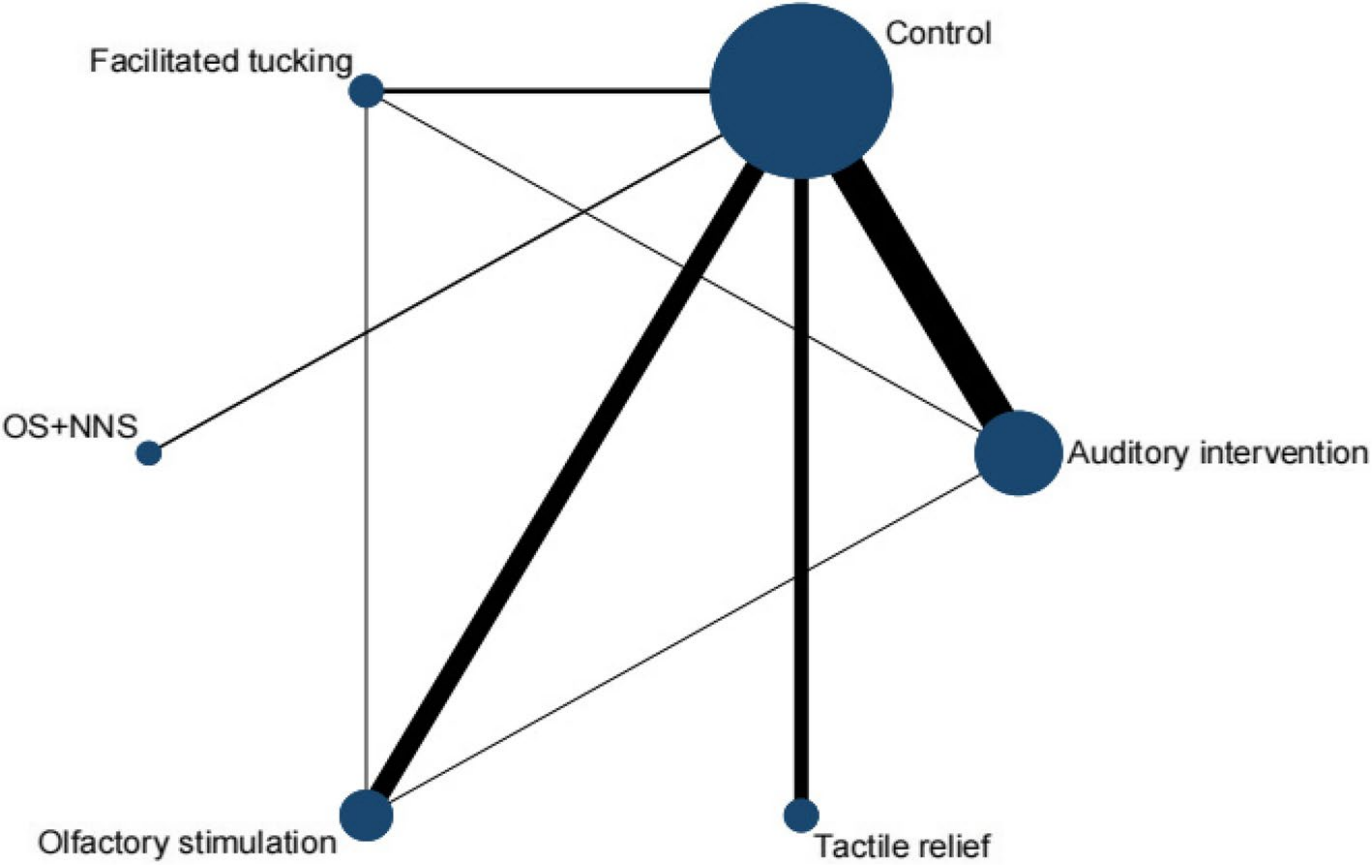
Network evidence diagram (oxygen saturation)

A total of 14 RCTs [32, 33, 38, 39, 41, 43, 47–50, 55, 56, 60, 63] were included in this meta-analysis by oxygen saturation, involving 5 interventions. Olfactory stimulation (3 RCTs), OS + NNS (2 RCTs), facilitated tucking (2 RCTs), auditory intervention (6 RCTs), and tactile relief (3 RCTs) were included. A total of 6 nodes were included in this meta-analysis, with each node representing an intervention or control (Fig. 5). The nodes with more significant interactions were control (16 interactions), auditory intervention (8 interactions), olfactory stimulation (5 interactions), and facilitated tucking (5 interactions). The results of the consistency analysis was shown in Additional file 1: Appendix Table 6. The results of the heterogeneity test indicated a high degree of heterogeneity with an I^2^ value of 81.5%.

**Fig. 6:**
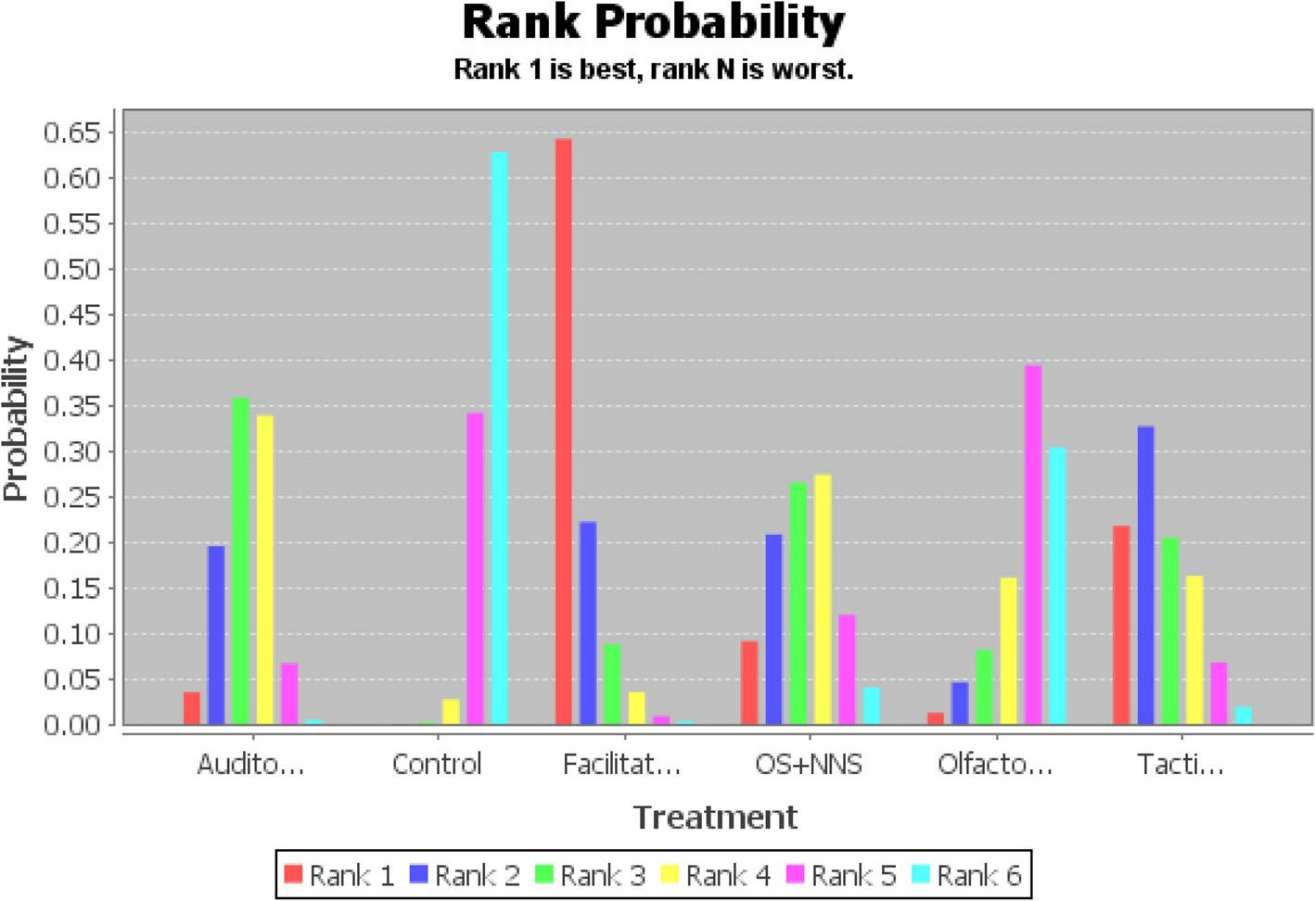
Probability ranking diagram of best interventions (oxygen saturation)

Based on moderate-quality evidence (Additional file 1: Appendix Table 9), facilitated tucking had the greatest SUCRA score, followed by tactile relief, auditory intervention, OS + NNS, olfactory stimulation, and control group (Fig. 6 and Additional file 1: Appendix Table 7). Compared to the control group, facilitated tucking was 1.94 (95% CI: 0.66,3.35, SUCRA score: 0.64) higher, the auditory intervention was 1.04 (95% CI: 0.22,2.04, SUCRA score: 0.36) higher, and the remaining comparisons were not statistically significant (Table 3). Therefore, the complete probability ranking was facilitated tucking (64%) > auditory intervention (36%) > control (63%) (Additional file 1: Appendix Table 7). The stability and credibility of the results were demonstrated in Additional file 1: Appendix Table 8 through the sensitivity analysis.

**Table 3:** The results of network meta-analysis (consistency model, oxygen saturation)

Auditory intervention	-1.04 (-2.04, -0.22)	0.89 (-0.62, 2.32)	0.00 (-1.66, 1.62)	-0.75 (-2.08, 0.86)	0.34 (-1.39, 1.91)
1.04 (0.22, 2.04)	Control	1.94 (0.66, 3.35)	1.04 (-0.24, 2.52)	0.30 (-0.95, 1.96)	1.38 (0.00, 2.80)
-0.89 (-2.32, 0.62)	-1.94 (-3.35, -0.66)	Facilitated tucking	-0.90 (-2.78, 1.05)	-1.64 (-3.15, 0.15)	-0.57 (-2.54, 1.37)
-0.00 (-1.62, 1.66)	-1.04 (-2.52, 0.24)	0.90 (-1.05, 2.78)	OS + NNS	-0.75 (-2.55, 1.34)	0.33 (-1.67, 2.23)
0.75 (-0.86, 2.08)	-0.30 (-1.96, 0.95)	1.64 (-0.15, 3.15)	0.75 (-1.34, 2.55)	Olfactory stimulation	1.06(-1.01, 2.91)
-0.34 (-1.91, 1.39)	-1.38 (-2.80, -0.00)	0.57 (-1.37, 2.54)	-0.33 (-2.23, 1.67)	-1.06 (-2.91, 1.01)	Tactile relief

### Heart rate

**Fig. 7:**
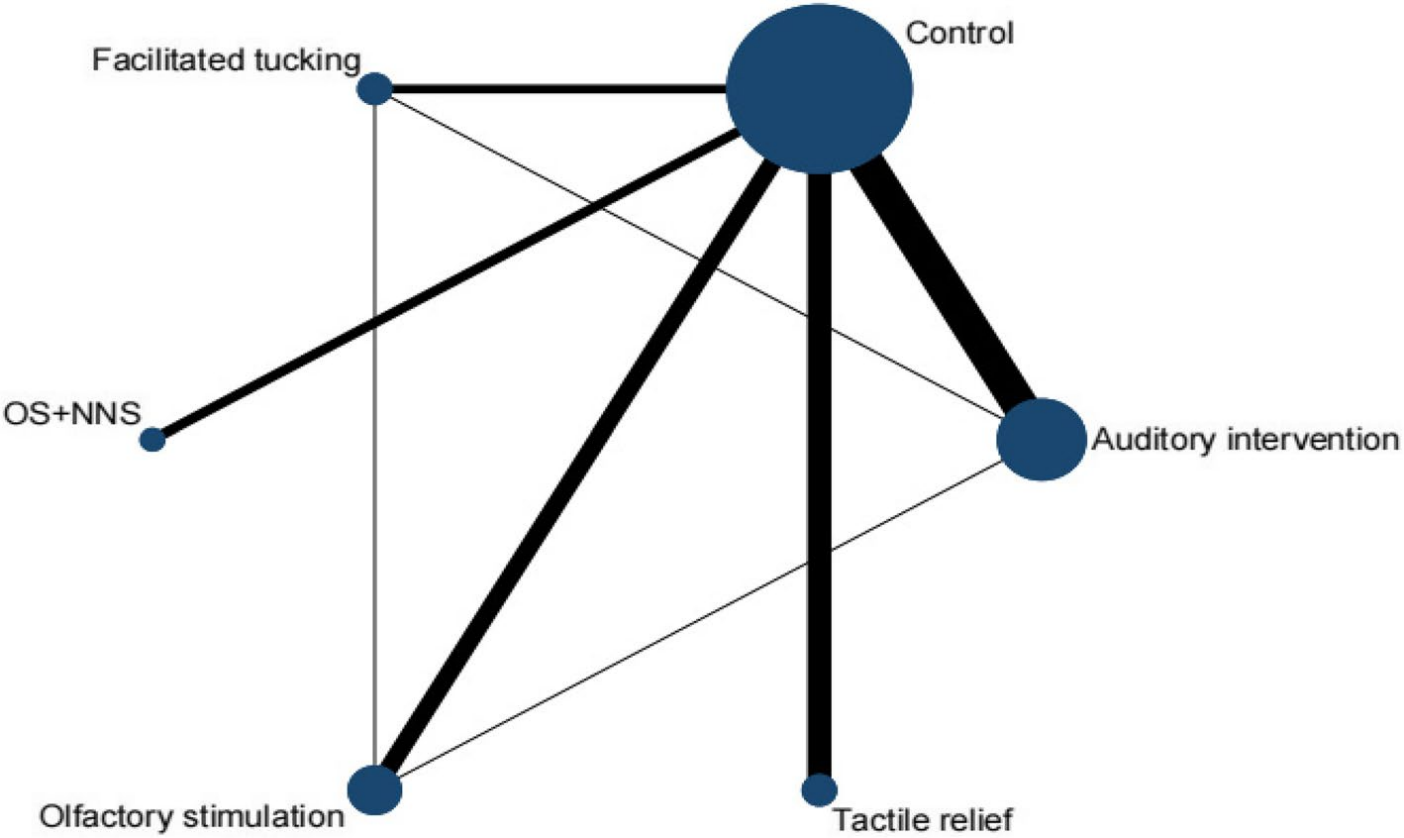
Network evidence diagram (heart rate)

A total of 14 RCTs [32, 33, 38, 39, 41, 43, 47–50, 55, 56, 60, 63] were included in this meta-analysis by heart rate, involving 5 interventions. Olfactory stimulation (3 RCTs), OS + NNS (2 RCTs), facilitated tucking (2 RCTs), auditory intervention (6 RCTs), and tactile relief (3 RCTs) were included. A total of 6 nodes were included in this meta-analysis, with each node representing an intervention or control (Fig. 7). The nodes with more significant interactions were control (16 interactions), auditory intervention (8 interactions), olfactory stimulation (5 interactions), and facilitated tucking (5 interactions). The results of the consistency analysis was shown in Additional file 1: Appendix Table 10. The results of the heterogeneity test indicated a high degree of heterogeneity with an I^2^ value of 99.3%.

**Fig. 8:**
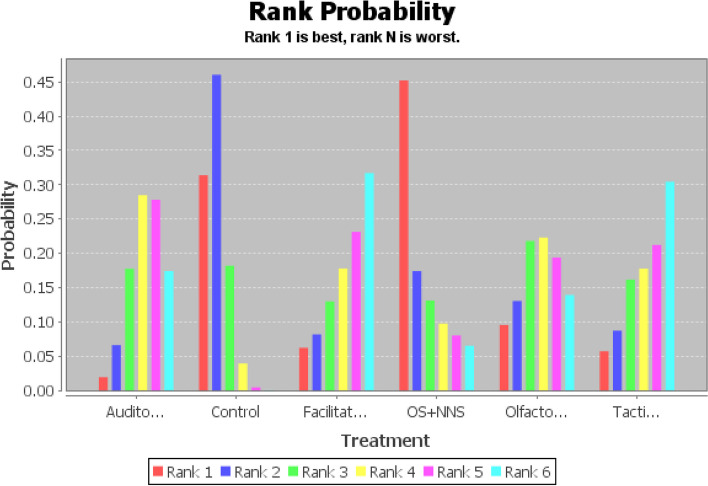
Probability ranking diagram of best interventions (heart rate)

Based on moderate-quality evidence (Additional file 1: Appendix Table 13), OS + NNS had the greatest SUCRA score, followed by control group, olfactory stimulation, auditory intervention, facilitated tucking, and tactile relief (Fig. 8 and Additional file 1: Appendix Table 11). However, this study found that there were no statistical differences between the interventions based on the data in Table 4. Therefore, the probability ranking analysis shown in Additional file 1: Appendix Table 11 was invalid. The stability and credibility of the results were demonstrated in Additional file 1: Appendix Table 12 through the sensitivity analysis.

**Table 4:** The results of network meta-analysis (consistency model, heart rate)

Auditory intervention	6.63 (-1.51, 15.03)	-0.94 (-15.22, 13.49)	6.86 (-9.77, 23.62)	1.50 (-11.14, 15.07)	-0.70 (-15.22, 14.39)
-6.63 (-15.03, 1.51)	Control	-7.62 (-20.63, 5.55)	0.14 (-14.41, 14.89)	-5.08 (-16.46, 6.11)	-7.20 (-19.54, 4.63)
0.94 (-13.49, 15.22)	7.62 (-5.55, 20.63)	Facilitated tucking	7.74 (-11.61, 27.44)	2.45 (-12.94, 18.37)	0.44 (-17.73, 18.14)
-6.86 (-23.62, 9.77)	-0.14 (-14.89, 14.41)	-7.74 (-27.44, 11.61)	OS + NNS	-5.11 (-23.76, 13.10)	-7.39 (-26.57, 11.60)
-1.50 (-15.07, 11.14)	5.08 (-6.11, 16.46)	-2.45 (-18.37, 12.94)	5.11 (-13.10, 23.76)	Olfactory stimulation	-2.12 (-18.71, 14.29)
0.70 (-14.39, 15.22)	7.20 (-4.63, 19.54)	-0.44 (-18.14, 17.73)	7.39 (-11.60, 26.57)	2.12 (-14.29, 18.71)	Tactile relief

## Discussion

This study conducted a network meta-analysis of 35 RCTs consisting of 2134 preterm infants to compare the efficacy of different interventions for pain relief. The results showed that in addition to tactile relief, interventions such as OS + NNS, facilitated tucking, auditory intervention, olfactory stimulation, and mixed intervention were significantly more effective in reducing pain compared to the control group. Among these interventions, OS + NNS was relatively more effective, while the mixed intervention was relatively less effective. In addition to analyzing the effects of the interventions on pain scores, the study also investigated their effects on oxygen saturation and heart rate. The results showed that only facilitated tucking and auditory intervention had a statistically significant improvement in oxygen saturation compared to the control group. However, none of the interventions exerted a significant effect on heart rate.

Research studies have indicated that multiple acute pain procedures in NICUs can result in “a chronically painful state” for preterm infants [64, 65], which is detrimental to pain recovery, neurodevelopment, and psychosocial health [66]. Non-pharmacological interventions for managing pain have gained significant attention in recent years. However, the effectiveness of these interventions has not yet been clear and remains an area of ongoing research [66, 67]. Based on this, this meta-analysis compared their efficacy and found that OS + NNS, facilitated tucking and auditory intervention were relatively more effective. According to the results of network meta-analysis, this study evaluated the combined intervention of OS + NNS. However, the role of OS + NNS in reducing pain among preterm infants did not reach a consensus. A systematic review [68] investigating the impact of non-pharmacological analgesic interventions during heel prick showed that OS + NNS did not significantly reduce pain scores, oxygen saturation, and heart rate. The difference in results may be due to variations in concentration and dose of sucrose solution used, as has been observed in other studies [69–72]. Subsequent studies can compare varying concentrations and dosages of sucrose solutions to more comprehensively observe their impact on pain levels among preterm infants.

Overall, the role of facilitated tucking in reducing pain scores and improving oxygen saturation was more pronounced. A systematic review [73] of the effects of different body positions on procedural pain in NICUs indicated that facilitating tucking by parents for at least 30 min was optimal for alleviating pain and stabilizing physiological, hormonal, and behavioral responses of the newborns. Notably, this meta-analysis only focused on facilitated tucking and did not include other postural interventions. A recent review [74] found that 7 different modified positions have positive effects on sleep, flexion maintenance, and pain management in preterm infants. This indicates that facilitated tucking is not necessarily the best position to reduce pain, and the efficacy and safety of different positions should be analyzed according to their different physiological conditions.

The third ranked non-pharmacological intervention was auditory intervention, which involves exposing preterm infants to sound stimulation through audio playback of white noise, mother's voice, lullabies, and maternal heart sounds [29, 38, 47, 49, 50, 60, 63]. Recent studies [18, 19] have demonstrated that this intervention has a positive impact on reducing pain levels, increasing comfort, improving physiological indicators, and promoting feeding outcomes among preterm infants. However, a systematic review [75] of 39 studies further differentiated auditory intervention, categorizing music-based interventions into music medicine and music therapy. Music medicine involves using recorded audio to stimulate preterm infants, while music therapy involves the use of live music interventions that are clinically and evidence-based and guided by a therapist. The results indicated that music medicine interventions were linked to pain relief, while music therapy had a beneficial impact on cardiac and respiratory function, as well as weight and eating behaviors. This indicates that the effects of the two auditory interventions are different, and subsequent studies can be discussed in this respect.

While traditional meta-analyses focus on comparing individual or the same category of interventions, this meta-analysis underwent a detailed search to systematically integrate published articles on pain management in preterm infants in English, and used a network meta-analysis to compare the relationship and efficacy between six non-pharmacological interventions. This study is closely relevant to clinical practice and may help medical professionals to adopt more effective interventions to reduce the pain and stress suffered from preterm infants during repeated painful procedures. In addition, this study recommends that clinical practitioners adopt a systematic, specialized, and multidisciplinary model for managing pain in preterm infants, paying attention to the combined effects of non-pharmacological interventions and their possible shortcomings in implementation.

Although this meta-analysis has provided some insights, it is crucial to recognize the limitations of this study. One of the major limitations was the high level of heterogeneity, which may be related to the differences in non-pharmacological interventions, the setting in which preterm infants were treated, variations in painful procedures, and the sample sizes used in the studies. Another was the publication bias suggested by funnel plots (Additional file 1: Appendix Figs. 1, 2 and 3), which may be relevant to the inclusion of several studies with poor study design or small scale in this meta-analysis. To increase the validity of these findings, it is necessary to conduct more high-quality studies.

## Conclusion

In conclusion, our study found that non-pharmacological interventions had different levels of efficacy in reducing pain scores and improving oxygen saturation, but no impact on heart rate was observed. Specifically, OS + NNS was found to be the relatively more effective intervention in reducing pain for preterm infants, while facilitated tucking was the relatively more effective in improving oxygen saturation. In addition, we hope for the development of more non-pharmacological interventions to ease the pain experienced by preterm infants during painful procedures, and we also aspire that our study can offer some support to clinical practices.

### Supplementary Information

**Additional file 1.**

## Data Availability

All data used in this review are derived from published studies.
