# Supplementary material for: Effect of non-pharmacological interventions on pain in preterm infants in the neonatal intensive care unit: a network meta-analysis of randomized controlled trials
Source: BMC Pediatr. 2024 Jan 3;24:9. doi: 10.1186/s12887-023-04488-y (PMC10765718; doi:10.1186/s12887-023-04488-y)
Supplement: Supplementary file 1 — Additional file 1. [file 12887_2023_4488_MOESM1_ESM.docx]

**Appendix Tables**

**Appendix Table 1: PubMed search strategy**

|  | Search equation | Results |
| --- | --- | --- |
| 1 | "Pain"[MH] OR "Pain Management"[MH] OR "Pain Measurement"[MH] OR "Pain, Procedural"[MH] | 495,299 |
| 2 | pain*[TIAB] OR cry*[TIAB] OR suffer*[TIAB] OR sob*[TIAB] OR weep*[TIAB] OR ache*[TIAB] OR aching[TIAB] OR agony[TIAB] OR afflict*[TIAB] OR anguish*[TIAB] OR sore*[TIAB] OR torment[TIAB] OR twinge*[TIAB] | 1,228,824 |
| 3 | "Infant"[MH] OR "Infant, Premature"[MH] OR "Intensive Care, Neonatal"[MH] OR "Intensive Care Units, Neonatal"[MH] | 1,243,292 |
| 4 | preterm[TIAB] OR premature*[TIAB] OR newborn*[TIAB] OR neonat*[TIAB] | 614,860 |
| 5 | "randomized controlled trial"[PT] OR "controlled clinical trial"[PT] OR randomized[TIAB] OR placebo[TIAB] OR randomly[TIAB] OR trial[TIAB] OR groups[TIAB] | 3,660,933 |
| 6 | (#1 OR #2) AND (#3 OR #4) AND #5 | 10,997 |
| 7 | #6 AND 2000-2023[Results By Year] AND Randomized Controlled Trial[Article Type] AND English[Language] | 2,238 |

**Appendix Table 2: The results of consistency analysis (PIPP score).**

| **Name** | **Direct Effect** | **Indirect Effect** | **Overall** | **P-Value** |
| --- | --- | --- | --- | --- |
| Auditory intervention vs Facilitated tucking | -0.30 (-4.11, 3.51) | -0.18 (-2.47, 2.13) | -0.04 (-2.03, 2.01) | 0.96 |
| Auditory intervention vs Mixed intervention | -1.18 (-5.28, 2.90) | 0.43 (-2.92, 3.74) | 0.22 (-2.33, 2.77) | 0.54 |
| Auditory intervention vs Olfactory stimulation | -0.25 (-3.01, 2.48) | 0.79 (-1.56, 3.05) | 0.68 (-1.19, 2.56) | 0.56 |
| Auditory intervention vs Tactile relief | -2.00 (-5.96, 1.84) | 1.97 (-0.27, 4.26) | 1.10 (-1.00, 3.22) | 0.07 |
| Facilitated tucking vs Olfactory stimulation | 0.90 (-2.91, 4.55) | 0.53 (-1.48, 2.51) | 0.70 (-1.15, 2.50) | 0.88 |
| Mixed intervention vs Tactile relief | -0.83 (-4.63, 2.99) | 1.90 (-0.76, 4.59) | 0.88 (-1.61, 3.32) | 0.23 |

**Appendix Table 3: Probability ranking analysis of best interventions (PIPP score).**

| Drug | Rank 1 | Rank 2 | Rank 3 | Rank 4 | Rank 5 | Rank 6 | Rank 7 |
| --- | --- | --- | --- | --- | --- | --- | --- |
| Auditory intervention | 0.00 | 0.05 | 0.12 | 0.20 | 0.27 | 0.27 | 0.09 |
| Control | 0.94 | 0.05 | 0.00 | 0.00 | 0.00 | 0.00 | 0.00 |
| Facilitated tucking | 0.00 | 0.05 | 0.12 | 0.20 | 0.27 | 0.29 | 0.07 |
| Mixed intervention | 0.02 | 0.14 | 0.18 | 0.18 | 0.18 | 0.21 | 0.09 |
| OS+NNS | 0.00 | 0.01 | 0.02 | 0.04 | 0.06 | 0.14 | 0.73 |
| Olfactory stimulation | 0.00 | 0.22 | 0.31 | 0.25 | 0.14 | 0.06 | 0.01 |
| Tactile relief | 0.03 | 0.47 | 0.26 | 0.13 | 0.07 | 0.03 | 0.00 |

**Appendix Table 4: The results of the sensitivity analysis (PIPP score).**

| Name | Consistency model | Inconsistency model |
| --- | --- | --- |
| Auditory intervention vs Control | -2.48 (-4.10, -0.91) | -3.05 (-5.10, -1.20) |
| Auditory intervention vs Facilitated tucking | 0.04 (-2.01, 2.03) | 0.21 (-2.28, 2.82) |
| Auditory intervention vs Mixed intervention | -0.22 (-2.77, 2.33) | 0.29 (-2.62, 3.34) |
| Auditory intervention vs OS+NNS | 1.45 (-1.31, 4.16) | 0.81 (-2.23, 3.65) |
| Control vs Mixed intervention | 2.26 (0.10, 4.38) | 2.37 (0.20, 4.48) |
| Facilitated tucking vs OS+NNS | 1.41 (-1.23, 4.02) | 1.44 (-1.16, 3.98) |

**Appendix Table 5: The results of the certainty of evidence using CINEMA framework (PIPP score).**

| **Comparison** | **N. of studies** | **Within-study bias** | **Reporting bias** | **Indirectness** | **Imprecision** | **Heterogeneity** | **Incoherence** | **Confidence rating** |
| --- | --- | --- | --- | --- | --- | --- | --- | --- |
| Auditory intervention:Control | 5 | No concerns | Low risk | No concerns | No concerns | Major concerns | No concerns | Moderate |
| Auditory intervention:Facilitated tucking | 1 | No concerns | Low risk | No concerns | Major concerns | No concerns | No concerns | Moderate |
| Auditory intervention:Mixed intervention | 1 | No concerns | Low risk | No concerns | Major concerns | No concerns | No concerns | Moderate |
| Auditory intervention:Olfactory stimulation | 2 | No concerns | Low risk | No concerns | Major concerns | No concerns | No concerns | Moderate |
| Auditory intervention:Tactile relief | 1 | No concerns | Low risk | No concerns | Major concerns | No concerns | Major concerns | Moderate |
| Control:Facilitated tucking | 8 | No concerns | Low risk | No concerns | No concerns | Major concerns | No concerns | Moderate |
| Control:Mixed intervention | 3 | No concerns | Low risk | No concerns | No concerns | Major concerns | No concerns | Moderate |
| Control:OS+NNS | 3 | No concerns | Low risk | No concerns | No concerns | Major concerns | Some concerns | Moderate |
| Control:Olfactory stimulation | 8 | No concerns | Low risk | No concerns | No concerns | Major concerns | No concerns | Moderate |
| Control:Tactile relief | 7 | Some concerns | Low risk | No concerns | No concerns | Major concerns | No concerns | Moderate |
| Facilitated tucking:Olfactory stimulation | 1 | No concerns | Low risk | No concerns | Major concerns | No concerns | No concerns | Moderate |
| Mixed intervention:Tactile relief | 1 | No concerns | Low risk | No concerns | Major concerns | No concerns | No concerns | Moderate |
| Auditory intervention:OS+NNS | 0 | No concerns | Low risk | No concerns | Major concerns | No concerns | Some concerns | Moderate |
| Facilitated tucking:Mixed intervention | 0 | No concerns | Low risk | No concerns | Major concerns | No concerns | Some concerns | Moderate |
| Facilitated tucking:OS+NNS | 0 | No concerns | Low risk | No concerns | Major concerns | No concerns | Some concerns | Moderate |
| Facilitated tucking:Tactile relief | 0 | No concerns | Low risk | No concerns | Major concerns | No concerns | Some concerns | Moderate |
| Mixed intervention:OS+NNS | 0 | No concerns | Low risk | No concerns | Major concerns | No concerns | Some concerns | Moderate |
| Mixed intervention:Olfactory stimulation | 0 | No concerns | Low risk | No concerns | Major concerns | No concerns | Some concerns | Moderate |
| Olfactory stimulation:OS+NNS | 0 | No concerns | Low risk | No concerns | Major concerns | No concerns | Some concerns | Moderate |
| OS+NNS:Tactile relief | 0 | No concerns | Low risk | No concerns | No concerns | Major concerns | Some concerns | Moderate |
| Olfactory stimulation:Tactile relief | 0 | No concerns | Low risk | No concerns | Major concerns | No concerns | Some concerns | Moderate |

**Appendix Table 6: The results of consistency analysis (oxygen saturation).**

| Name | Direct Effect | Indirect Effect | Overall | P-Value |
| --- | --- | --- | --- | --- |
| Auditory intervention vs Facilitated tucking | 1.12 (-0.93, 3.11) | 0.74 (-2.03, 3.42) | 0.89 (-0.62, 2.32) | 0.80 |
| Auditory intervention vs Olfactory stimulation | -0.75 (-2.56, 1.01) | 0.71 (-2.09, 3.57) | -0.75 (-2.08, 0.86) | 0.36 |
| Facilitated tucking vs Olfactory stimulation | -1.91 (-3.69, -0.01) | -0.27 (-3.14, 2.72) | -1.64 (-3.15, 0.15) | 0.32 |

**Appendix Table 7: Probability ranking analysis of best interventions (oxygen saturation).**

| Drug | Rank 1 | Rank 2 | Rank 3 | Rank 4 | Rank 5 | Rank 6 |
| --- | --- | --- | --- | --- | --- | --- |
| Auditory intervention | 0.04 | 0.20 | 0.36 | 0.34 | 0.07 | 0.00 |
| Control | 0.00 | 0.00 | 0.00 | 0.03 | 0.34 | 0.63 |
| Facilitated tucking | 0.64 | 0.22 | 0.09 | 0.04 | 0.01 | 0.00 |
| OS+NNS | 0.09 | 0.21 | 0.27 | 0.27 | 0.12 | 0.04 |
| Olfactory stimulation | 0.01 | 0.05 | 0.08 | 0.16 | 0.39 | 0.30 |
| Tactile relief | 0.22 | 0.33 | 0.20 | 0.16 | 0.07 | 0.02 |

**Appendix Table 8: The results of the sensitivity analysis (oxygen saturation).**

| Name | Consistency model | Inconsistency model |
| --- | --- | --- |
| Auditory intervention vs Facilitated tucking | -0.89 (-2.32, 0.62) | -1.04 (-2.56, 0.55) |
| Auditory intervention vs Olfactory stimulation | 0.75 (-0.86, 2.08) | 0.71 (-1.00, 2.24) |
| Facilitated tucking vs Olfactory stimulation | 1.64 (-0.15, 3.15) | 1.76 (-0.02, 3.27) |

**Appendix Table 9: The results of the certainty of evidence using CINEMA framework (oxygen saturation).**

| **Comparison** | **N. of studies** | **Within-study bias** | **Reporting bias** | **Indirectness** | **Imprecision** | **Heterogeneity** | **Incoherence** | **Confidence rating** |
| --- | --- | --- | --- | --- | --- | --- | --- | --- |
| Auditory intervention:Control | 6 | No concerns | Low risk | No concerns | No concerns | Major concerns | No concerns | Moderate |
| Auditory intervention:Facilitated tucking | 1 | No concerns | Low risk | No concerns | Major concerns | No concerns | No concerns | Moderate |
| Auditory intervention:Olfactory stimulation | 1 | No concerns | Low risk | No concerns | Major concerns | No concerns | No concerns | Moderate |
| Control:Facilitated tucking | 2 | No concerns | Low risk | No concerns | No concerns | Major concerns | No concerns | Moderate |
| Control:OS+NNS | 2 | Some concerns | Low risk | No concerns | Major concerns | No concerns | No concerns | Moderate |
| Control:Olfactory stimulation | 3 | No concerns | Low risk | No concerns | Major concerns | No concerns | No concerns | Moderate |
| Control:Tactile relief | 3 | No concerns | Low risk | No concerns | No concerns | Major concerns | No concerns | Moderate |
| Facilitated tucking:Olfactory stimulation | 1 | No concerns | Low risk | No concerns | No concerns | Major concerns | No concerns | Moderate |
| Auditory intervention:OS+NNS | 0 | No concerns | Low risk | No concerns | Major concerns | No concerns | No concerns | Moderate |
| Auditory intervention:Tactile relief | 0 | No concerns | Low risk | No concerns | Major concerns | No concerns | No concerns | Moderate |
| Facilitated tucking:OS+NNS | 0 | No concerns | Low risk | No concerns | Major concerns | No concerns | No concerns | Moderate |
| Facilitated tucking:Tactile relief | 0 | No concerns | Low risk | No concerns | Major concerns | No concerns | No concerns | Moderate |
| Olfactory stimulation:OS+NNS | 0 | No concerns | Low risk | No concerns | Major concerns | No concerns | No concerns | Moderate |
| OS+NNS:Tactile relief | 0 | No concerns | Low risk | No concerns | Major concerns | No concerns | No concerns | Moderate |
| Olfactory stimulation:Tactile relief | 0 | No concerns | Low risk | No concerns | Major concerns | No concerns | No concerns | Moderate |

**Appendix Table 10: The results of consistency analysis (heart rate).**

| Name | Direct Effect | Indirect Effect | Overall | P-Value |
| --- | --- | --- | --- | --- |
| Auditory intervention vs Facilitated tucking | -9.49 (-29.82, 10.49) | 3.31 (-20.89, 26.50) | -0.94 (-15.22, 13.49) | 0.40 |
| Auditory intervention vs Olfactory stimulation | -6.81 (-27.27, 13.62) | 3.56 (-13.92, 20.96) | 1.50 (-11.14, 15.07) | 0.41 |
| Facilitated tucking vs Olfactory stimulation | 2.60 (-17.48, 23.08) | 4.67 (-16.44, 25.30) | 2.45 (-12.94, 18.37) | 0.90 |

**Appendix Table 11: Probability ranking analysis of best interventions (heart rate).**

| Drug | Rank 1 | Rank 2 | Rank 3 | Rank 4 | Rank 5 | Rank 6 |
| --- | --- | --- | --- | --- | --- | --- |
| Auditory intervention | 0.02 | 0.07 | 0.18 | 0.28 | 0.28 | 0.17 |
| Control | 0.31 | 0.46 | 0.18 | 0.04 | 0.00 | 0.00 |
| Facilitated tucking | 0.06 | 0.08 | 0.13 | 0.18 | 0.23 | 0.32 |
| OS+NNS | 0.45 | 0.17 | 0.13 | 0.10 | 0.08 | 0.07 |
| Olfactory stimulation | 0.10 | 0.13 | 0.22 | 0.22 | 0.19 | 0.14 |
| Tactile relief | 0.06 | 0.09 | 0.16 | 0.18 | 0.21 | 0.30 |

**Appendix Table 12: The results of the sensitivity analysis (heart rate).**

| Name | Consistency model | Inconsistency model |
| --- | --- | --- |
| Auditory intervention vs Facilitated tucking | 0.94 (-13.49, 15.22) | 4.42 (-11.93, 20.13) |
| Auditory intervention vs Olfactory stimulation | -1.50 (-15.07, 11.14) | 1.98 (-13.86, 17.96) |
| Facilitated tucking vs Olfactory stimulation | -2.45 (-18.37, 12.94) | -2.33 (-18.46, 13.39) |

**Appendix Table 13: The results of the certainty of evidence using CINEMA framework (heart rate).**

| **Comparison** | **N. of studies** | **Within-study bias** | **Reporting bias** | **Indirectness** | **Imprecision** | **Heterogeneity** | **Incoherence** | **Confidence rating** |
| --- | --- | --- | --- | --- | --- | --- | --- | --- |
| Auditory intervention:Control | 6 | No concerns | Low risk | No concerns | Major concerns | No concerns | No concerns | Moderate |
| Auditory intervention:Facilitated tucking | 1 | No concerns | Low risk | No concerns | Major concerns | No concerns | No concerns | Moderate |
| Auditory intervention:Olfactory stimulation | 1 | No concerns | Low risk | No concerns | Major concerns | No concerns | No concerns | Moderate |
| Control:Facilitated tucking | 2 | No concerns | Low risk | No concerns | Major concerns | No concerns | No concerns | Moderate |
| Control:OS+NNS | 2 | No concerns | Low risk | No concerns | Major concerns | No concerns | No concerns | Moderate |
| Control:Olfactory stimulation | 3 | No concerns | Low risk | No concerns | Major concerns | No concerns | No concerns | Moderate |
| Control:Tactile relief | 3 | No concerns | Low risk | No concerns | Major concerns | No concerns | No concerns | Moderate |
| Facilitated tucking:Olfactory stimulation | 1 | No concerns | Low risk | No concerns | Major concerns | No concerns | No concerns | Moderate |
| Auditory intervention:OS+NNS | 0 | No concerns | Low risk | No concerns | Major concerns | No concerns | No concerns | Moderate |
| Auditory intervention:Tactile relief | 0 | No concerns | Low risk | No concerns | Major concerns | No concerns | No concerns | Moderate |
| Facilitated tucking:OS+NNS | 0 | No concerns | Low risk | No concerns | Major concerns | No concerns | No concerns | Moderate |
| Facilitated tucking:Tactile relief | 0 | No concerns | Low risk | No concerns | Major concerns | No concerns | No concerns | Moderate |
| Olfactory stimulation:OS+NNS | 0 | No concerns | Low risk | No concerns | Major concerns | No concerns | No concerns | Moderate |
| OS+NNS:Tactile relief | 0 | No concerns | Low risk | No concerns | Major concerns | No concerns | No concerns | Moderate |
| Olfactory stimulation:Tactile relief | 0 | No concerns | Low risk | No concerns | Major concerns | No concerns | No concerns | Moderate |

**Appendix Figures**

**Appendix Figure 1: Funnel plot for assessing publication biases (PIPP score).**


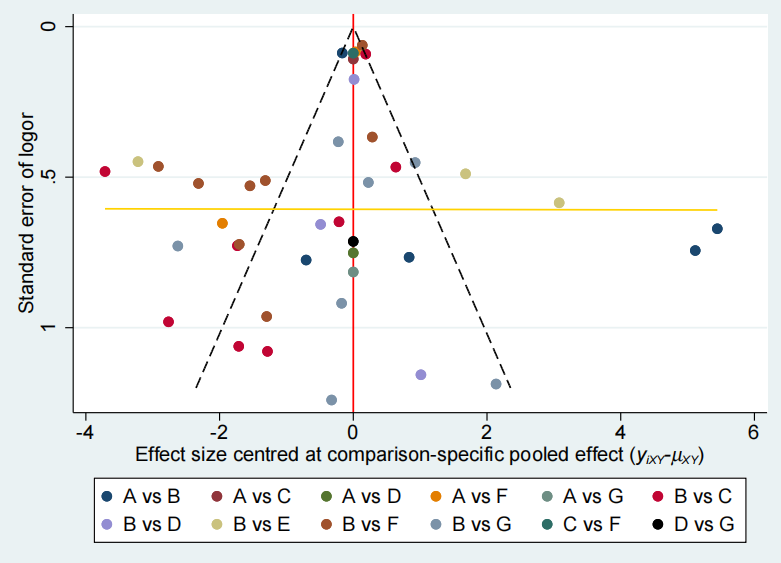


A: Auditory intervention; B: Control; C: Facilitated tucking; D: Mixed intervention; E: OS+NNS; F: Olfactory stimulation; G: Tactile relief

**Appendix Figure 2: Funnel plot for assessing publication biases (oxygen saturation).**


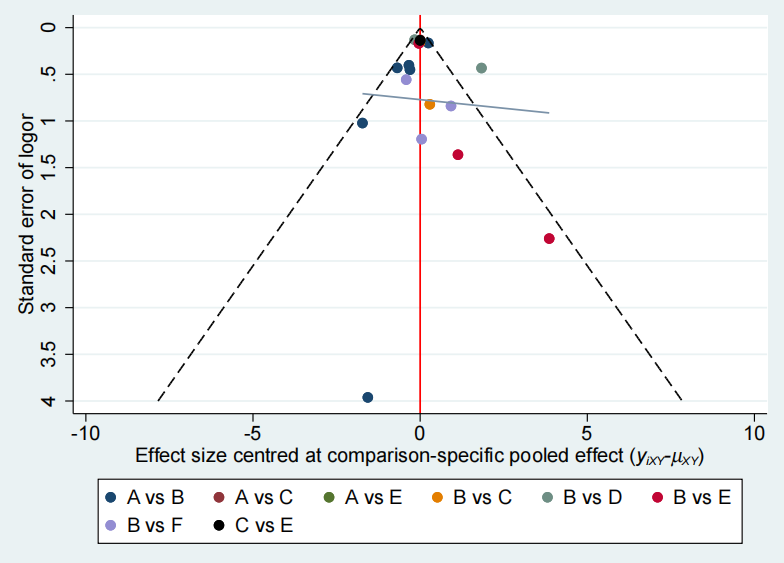


A: Auditory intervention; B: Control; C: Facilitated tucking; D: OS+NNS; E: Olfactory stimulation; F: Tactile relief

**Appendix Figure 3: Funnel plot for assessing publication biases (heart rate).**


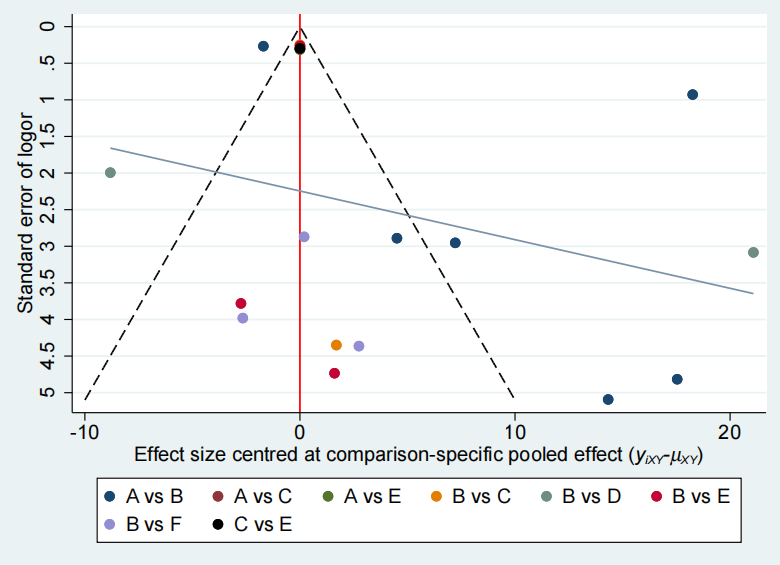


A: Auditory intervention; B: Control; C: Facilitated tucking; D: OS+NNS; E: Olfactory stimulation; F: Tactile relief
